# Neurotoxicity of Broad‐Spectrum Antibiotics in Urinary Tract Infections: Mechanisms and Clinical Implications

**DOI:** 10.1155/aiu/1858698

**Published:** 2026-10-17

**Authors:** Lama Hachem, Batoul Darwish, Maram Chaaban, Rawan Khadra, Hashem Shehade, Antoine Abou Fayyad, Hisham F. Bahmad, Georges Daoud, Wassim Abou-Kheir

**Affiliations:** ^1^ Department of Anatomy, Cell Biology and Physiological Sciences Faculty of Medicine American University of Beirut Beirut 1107-2020 Lebanon aub.edu.lb; ^2^ Department of Experimental Pathology, Immunology and Microbiology Faculty of Medicine American University of Beirut Beirut 1107-2020 Lebanon aub.edu.lb; ^3^ Department of Pathology and Laboratory Medicine University of Miami Miller School of Medicine Miami Florida 33136 USA miami.edu

**Keywords:** antibiotics, broad-spectrum, CNS, mechanisms, neurotoxicity, UTI

## Abstract

Urinary tract infections (UTIs) remain among the most common bacterial infections worldwide, affecting millions every year. While *Escherichia coli* (*E*. *coli*) is the predominant pathogen, a broad range of Gram‐negative and Gram‐positive organisms can be implicated. Treating UTIs, particularly complicated or severe infections, frequently relies on broad‐spectrum antibiotics, often started empirically before identifying the causative pathogen. However, despite their ability to cover a wide spectrum of pathogens, they have become a growing threat to public health, mainly due to the rise of antimicrobial resistance, but also due to the many other adverse effects they can potentially cause. Neurotoxicity represents one of the most consequential yet underrecognized complications of antibiotic therapy. Broad‐spectrum agents commonly used for UTIs, including fosfomycin, fluoroquinolones, carbapenems, and cephalosporins, have been associated with a wide range of neurologic manifestations such as epileptic seizures, encephalopathy, movement disorders, delirium, and psychosis, varying in onset, duration, severity, and reversibility. To induce such reactions, antibiotics interact with the central nervous system (CNS) in several ways that can threaten its integrity and disrupt homeostatic control of its components. This review provides a synopsis of the known neurological adverse reactions linked to four major antibiotic classes routinely used in UTI management and the mechanistic pathways underlying CNS toxicity. An overall clearer understanding of these risks may help clinicians balance therapeutic benefit against potential harm, optimize antimicrobial selection, and improve prevention and management strategies for antibiotic‐associated neurotoxicity.

## 1. Introduction

### 1.1. Urinary Tract Infections (UTIs)

UTIs are among the most prevalent bacterial infections globally, affecting an estimated 150 million people annually [1] with a markedly higher incidence in women due to anatomic and physiologic factors [2]. The most common bacteria known to cause UTIs is *Escherichia coli* (*E*. *coli*), but *Klebsiella pneumoniae* (*K. pneumoniae*), *Proteus mirabilis* (*P. mirabilis*), *Enterococcus faecalis* (*E. faecalis*), *Staphylococcus saprophyticus* (*S. saprophyticus*), and *Pseudomonas aeruginosa* (*P. aeruginosa*) also contribute substantially to disease burden.

UTIs are classified according to the involved anatomical site (urethritis, cystitis, ureteritis, or pyelonephritis) and clinically categorized as uncomplicated or complicated. Complicated UTIs usually occur in individuals with functional or structural abnormalities of the urinary tract, compromised immune system, comorbidities such as diabetes mellitus, or pregnancy. Besides, UTIs in men are typically classified as complicated due to frequent prostatic involvement. Infections in otherwise healthy, nonpregnant women with normal urinary tracts are considered uncomplicated.

### 1.2. Antimicrobial Therapy in the Treatment of UTIs

Management of uncomplicated UTIs generally relies on first‐line therapies such as fosfomycin tromethamine, nitrofurantoin, pivmecillinam, or trimethoprim–sulfamethoxazole. Fluoroquinolones and β‐lactams are reserved as second‐line agents due to concerns regarding resistance and side effects [1].

In contrast, complicated UTIs require prompt antimicrobial coverage to prevent serious, life‐threatening complications such as sepsis or systemic dissemination. Therefore, empiric therapy with broad‐spectrum antibiotics is often initiated before pathogen identification, followed by de‐escalation to targeted therapy based on urine culture results [3]. β‐lactams, which include cephalosporins and carbapenems, are the cornerstone of empiric treatment in severe or nosocomial cases, while fluoroquinolones remain widely used due to their oral bioavailability and broad activity [4].

### 1.3. Antibiotic‐Induced Neurotoxicity: An Emerging Clinical Concern

Broad‐spectrum antibiotics are invaluable because of their activity against diverse Gram‐negative and Gram‐positive bacterial agents. However, over the years, resistance to these antimicrobials, as well as increasing reports of significant adverse drug reactions (ADRs) related to their use, have become a serious threat to public health and have highlighted the importance of seeking more optimized and safer alternatives to their use. Antibiotic‐related ADRs account for approximately 5%–10% of hospital admissions, and 0.1%–0.3% of these cases can be life‐threatening. These include hematologic (e.g., leukopenia), pulmonary, renal, and gastrointestinal manifestations such as nausea, vomiting, and diarrhea.

Beyond these systemic adverse effects, antibiotic‐induced neurotoxicity has increasingly become a major problem associated with antimicrobial use. Neurological manifestations can occur during therapy or shortly after and can range from mild cognitive disturbances to severe manifestations such as seizures, encephalopathy, movement disorders, and neuropsychiatric symptoms. In 2023, a total of 14,407,157 FAERS (Food and Drug Administration [FDA] Adverse Event [AE] Reporting System) reports were analyzed to assess the risk of antibiotic‐induced seizures, revealing that 10 antibiotics are significantly associated with the risk of seizures [5]. The risk and severity of antibiotic‐induced neurotoxicity vary according to antibiotic class and patient‐specific susceptibility factors [6].

### 1.4. Antibiotic‐Induced Neurotoxicity in UTI Patients

Patients with UTIs represent a population at increased risk of antibiotic‐induced neurotoxicity due to the convergence of multiple predisposing factors rather than the infection itself. UTIs occur most frequently in older adults, individuals with chronic kidney disease, diabetes, urinary tract abnormalities, or those requiring recurrent antibiotic therapy. Many of these patients exhibit impaired renal function, which reduces the clearance of renally excreted antibiotics such as fluoroquinolones, cephalosporins, carbapenems, and aminoglycosides, leading to elevated systemic and central nervous system (CNS) drug exposure. Advanced age further increases susceptibility through age‐related changes in pharmacokinetics, blood–brain barrier (BBB) integrity, and neuronal reserve [7]. In addition, recurrent or complicated UTIs often necessitate prolonged or repeated courses of broad‐spectrum antibiotics, increasing cumulative neurotoxic risk. While severe systemic infections can contribute to neurological manifestations through inflammation, metabolic disturbances, or sepsis‐associated encephalopathy, uncomplicated UTIs are not themselves considered a primary cause of antibiotic‐induced neurotoxicity. Instead, the heightened vulnerability in this patient population arises predominantly from host‐related factors, particularly advanced age and impaired renal function, together with the pharmacokinetic properties of commonly prescribed antimicrobial agents. These factors may result in reduced drug clearance, systemic accumulation, and increased CNS exposure, thereby predisposing susceptible individuals to neurotoxic adverse effects [8]. Recognition of these interacting factors is essential for optimizing antimicrobial selection, implementing appropriate dose adjustments based on renal function, and ensuring careful neurological monitoring in patients at increased risk.

This review paper aims to investigate and characterize, through case reports and original studies, the neurotoxic manifestations associated with four broad‐spectrum antibiotic families commonly used in UTI treatment: fosfomycin, fluoroquinolones, carbapenems, and cephalosporins (Figure 1). Additionally, we examine the mechanistic pathways by which these agents interact with the CNS to promote neurotoxicity. Understanding these mechanisms is essential for informed risk–benefit assessment, as well as providing a gateway to find safer but equally effective treatment options for UTIs. By integrating clinical and mechanistic perspectives, this review aims to support improved risk stratification and more informed antibiotic selection in clinical practice.

**FIGURE 1:**
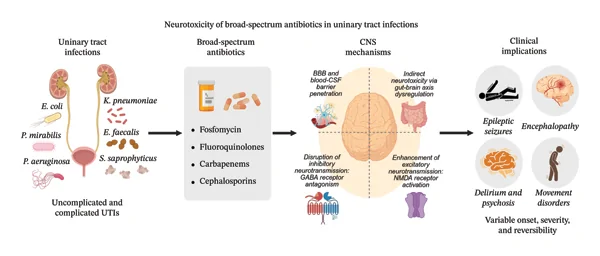
Neurotoxicity of broad‐spectrum antibiotics in urinary tract infections. Created with BioRender (2026).

## 2. Methods

### 2.1. Search Strategy

A systematic literature search was conducted in PubMed, Embase, the Cochrane Central Register of Controlled Trials (CENTRAL), and Google Scholar from database inception through June 2026. The search strategy incorporated both Medical Subject Headings (MeSH) and free‐text terms, combined using Boolean operators to maximize retrieval of relevant studies. Search terms included “antibiotics,” “neurotoxicity,” “antibiotic‐induced neurotoxicity,” “fosfomycin,” “Fluoroquinolones,” “Carbapenems,” “Cephalosporins,” “urinary tract infection,” “adverse drug reaction,” “UTI,” and “cystitis.” In addition, the reference lists of eligible publications were manually screened to identify further relevant studies that may not have been captured through the electronic database search.

### 2.2. Study Selection and Evidence Integration

Following the literature search, retrieved records were screened for relevance based on titles and abstracts, with full‐text articles assessed when necessary to determine eligibility. Studies were included if they provided relevant information on antibiotic‐associated neurotoxicity, particularly in the context of UTIs or the use of broad‐spectrum antibiotics commonly employed in its management. No restrictions were applied based on study design; both clinical and preclinical studies were considered, including clinical trials, observational studies, pharmacovigilance reports, review articles, and relevant experimental animal studies that contributed to the understanding of underlying neurotoxic mechanisms. Data from the included literature were extracted and synthesized narratively, with emphasis on clinical manifestations, mechanistic pathways, and risk factors associated with neurotoxicity. Given the heterogeneity of the available evidence, a qualitative synthesis approach was adopted, and no quantitative meta‐analysis was performed.

To address the heterogeneity of the underlying literature, each body of evidence discussed in this review was assigned to one of three levels, and this level is stated explicitly wherever a mechanism or a risk estimate is presented. Clinical evidence comprises randomized controlled trials, prospective and retrospective cohort studies, case–control studies, systematic reviews of such studies, and regulatory pharmacovigilance analyses; this evidence can support statements about incidence, risk factors, and prognosis in patients. Clinical descriptive evidence comprises case reports and case series, which establish that a phenomenon occurs and characterize its clinical phenotype, but which carry no denominator and therefore cannot support estimates of frequency or of relative risk. Preclinical evidence comprises in vitro receptor‐binding and cell‐culture studies and experimental animal work; this evidence can establish biological plausibility and identify candidate molecular mechanisms but cannot by itself demonstrate that a mechanism operates at clinically achievable drug exposures in patients. Where a mechanism is supported exclusively by preclinical data, this is stated in the text, and the evidence base underlying each proposed mechanism is summarized in Table 1.

**TABLE 1:** Strength of the evidence underlying each proposed mechanism of antibiotic‐induced neurotoxicity.

Mechanism	Preclinical evidence	Human/clinical evidence	Overall evidence level
CNS penetration across the BBB and blood–CSF barrier as a prerequisite for direct neurotoxicity	In vitro permeability and physicochemical studies; animal CSF pharmacokinetics	Human CSF: plasma pharmacokinetic studies for all four classes (no human data for ertapenem); measured CSF concentrations in patients with cefepime encephalopathy	Established in humans as a pharmacokinetic phenomenon; however, penetration does not predict the rank order of neurotoxicity between agents
GABA‐A receptor antagonism	Receptor‐binding assays; intracerebroventricular convulsant‐dose studies in rodents; structure–activity series (carbapenem C2 side chain, quinolone C7 substituent)	Indirect only: the clinical phenotype (seizures, myoclonus, nonconvulsive status epilepticus) and characteristic EEG patterns are consistent with loss of inhibitory tone; potentiation by concomitant NSAIDs is described clinically	Strong preclinical, indirect clinical. Reported in vitro IC50 values for some agents exceed clinically achievable CSF concentrations, an unresolved discrepancy
Glutamatergic/NMDA‐mediated excitotoxicity (fluoroquinolones)	In vitro rat hippocampal slice electrophysiology	None	Preclinical only
Mitochondrial dysfunction, oxidative stress, and neuroinflammation	Mammalian cell‐culture studies (superoxide, H2O2, γ‐H2AX, 8‐OHdG); rodent behavioral studies	None in human neurons	Preclinical only
Renal accumulation and excessive systemic exposure	Population pharmacokinetic modeling	Multiple cohorts with reproducible concentration–toxicity relationships and ROC‐derived thresholds; 80%–90% of cefepime cases have renal impairment; GFR shows a dose–response relationship with antibiotic‐associated encephalopathy at population level	Established in humans; the best‐supported clinical determinant
Host CNS vulnerability (preexisting CNS disease, prior seizures, advanced age)	Kindled‐mouse models	Independent risk factor in multivariable analyses across several cohorts	Established in humans
Indirect neurotoxicity via antibiotic‐induced gut dysbiosis and the gut–brain axis	Extensive: germ‐free and antibiotic‐depleted rodent models (behavior, neurogenesis, BDNF, tight junction gene expression, neurotransmitter turnover)	None. Human studies of antibiotic exposure and neuropsychiatric or cognitive outcomes are observational, inconsistent in direction, and did not measure the gut microbiota. No study has linked dysbiosis to neurological complications in patients treated for UTI	Preclinical only; hypothesis‐generating
Probiotic modulation as a mitigating strategy	Rodent studies of dysbiosis reversal	Clinical benefit demonstrated only for gastrointestinal endpoints (*C. difficile*–associated diarrhea) and only at high baseline risk. No trial of any neurological endpoint	Preclinical only for the neurological indication
Therapeutic drug monitoring as a preventive intervention	Population pharmacokinetic simulation of neurotoxicity probability	Randomized trials show improved target attainment but no demonstrated reduction in neurotoxicity; the only meta‐analytic estimate is imprecise (OR 0.86, 95% CI: 0.08–9.65) and derived from observational data	Mechanistically justified; interventional benefit unproven

*Note:* GABA, γ‐aminobutyric acid; IC50, half‐maximal inhibitory concentration.

Abbreviations: BBB, blood–brain barrier; BDNF, brain‐derived neurotrophic factor; CNS, central nervous system; CSF, cerebrospinal fluid; EEG, electroencephalography; GFR, glomerular filtration rate; NMDA, N‐methyl‐D‐aspartate; NSAID, nonsteroidal anti‐inflammatory drug; OR, odds ratio; ROC, receiver operating characteristic; UTI, urinary tract infection.

## 3. Adverse and Neurotoxic Effects of Broad‐Spectrum Antibiotics Used in UTI Management

### 3.1. Fosfomycin

Fosfomycin is a phosphonic acid derivative that constitutes a distinct class of antibiotics. It is a broad‐spectrum, bactericidal agent active against a wide range of Gram‐positive and Gram‐negative bacteria through irreversible inhibition of bacterial cell wall synthesis at an early stage. Fosfomycin enters bacterial cells via active transport systems and, once in the cytoplasm, inactivates enolpyruvyl transferase (MurA), a key enzyme involved in peptidoglycan biosynthesis, leading to bacterial cell death [9]. Owing to its unique chemical structure and mechanism of action, fosfomycin inhibits peptidoglycan synthesis at a stage prior to β‐lactams, minimizing the likelihood of cross‐resistance with other antimicrobial agents. As a result, fosfomycin has emerged as a promising candidate for treating systemic infections.

Clinically, fosfomycin is most commonly prescribed for the treatment of acute uncomplicated lower UTIs, particularly cystitis caused by *E. coli*. It is typically administered as a single oral dose of fosfomycin tromethamine, a formulation with enhanced oral bioavailability compared to other salts [10].

Given its single‐dose administration and favorable pharmacokinetic profile, fosfomycin is generally well tolerated. Reported adverse effects are usually mild and transient, predominantly involving gastrointestinal disturbances as shown in a FAERS analysis across oral fosfomycin trials (*n* = 28) in 2743 patients and parenteral fosfomycin trials (*n* = 23) in 1242 patients [11]. Neurotoxic manifestations are thus exceedingly rare and largely limited to nonspecific symptoms such as dizziness and headache [10]. In the pooled registration database of oral fosfomycin tromethamine (*n* = 1233), the only neurological drug‐related AEs reported at an incidence exceeding 1% were nervous‐system events of mild severity: headache (3.9%), dizziness (1.3%), and asthenia (1.1%). Serious neurotoxicity has not been established as an entity for this agent. A regulatory review combining the published literature with the FAERS—covering 2743 patients in oral comparative trials, 1242 patients in parenteral trials, and 559 spontaneous reports—identified disproportionality signals for hematological, hepatic, dermatological, and gastrointestinal events but none for any nervous‐system event [11]; a systematic review of 128 studies comprising 5527 patients likewise reported a favorable safety profile [12]; the randomized ZEUS trial of intravenous fosfomycin in complicated UTI and acute pyelonephritis (*n* = 464) recorded no seizures or encephalopathy in either arm [13]; and a real‐world cohort of 224 patients receiving intravenous fosfomycin, in which 42.4% experienced at least one AE, documented no neurological AEs at all [14]. It should nevertheless be emphasized that no denominator‐based estimate of serious fosfomycin neurotoxicity exists, so the apparent rarity of such events reflects both a genuinely low signal and the limits of what has been measured. However, emerging experimental evidence suggests that fosfomycin may exert subtle effects on CNS homeostasis. In a recent study evaluating the crosstalk between UTIs and hippocampal neurogenesis in rats, both UTI‐induced rats treated with fosfomycin and sham‐treated animals receiving fosfomycin demonstrated a significant reduction in BrdU‐positive cells within the dentate gyrus of the hippocampus, indicating decreased proliferation of neural stem cells compared with untreated controls [15]. Although these findings raise the possibility that fosfomycin may negatively influence hippocampal neurogenesis, this potential effect remains insufficiently characterized in the current literature and warrants further investigation.

### 3.2. Fluoroquinolones

Fluoroquinolones are broad‐spectrum antibiotics derived from the quinolone family and exhibit activity against a wide range of Gram‐negative and Gram‐positive organisms. The antibacterial effect is mediated through inhibition of bacterial DNA gyrase—key target in Gram‐negative bacteria—and topoisomerase IV—key target in Gram‐positive bacteria—thereby disrupting DNA replication and leading to bacterial cell death. Due to increasing concerns regarding resistance and adverse effects, fluoroquinolones are now recommended primarily as second‐line agents following failure of narrow‐spectrum antibiotics [16].

Despite these recommendations, fluoroquinolones remain frequently prescribed for UTIs, particularly complicated infections and acute pyelonephritis, as well as for gastrointestinal, skin, and soft tissue infections. Numerous studies have documented their neurotoxic potential, with reported manifestations including headache, confusion, tremor, psychosis, and seizures [17]. Estimates of how frequently these events occur vary by an order of magnitude according to the outcome studied and the design used, and it is important to distinguish them. Figures in the region of 1%–4.4% for neuropsychiatric adverse effects, with roughly 0.5% of patients developing severe or life‐threatening manifestations, are widely quoted [18], but these are derived from narrative syntheses without a stated denominator and should be regarded as an indicative literature range rather than an incidence. Where absolute risks have been calculated, they are considerably smaller. In a self‐controlled case‐series analysis of two national databases, the absolute risk of seizure was 0.72 per 10,000 fluoroquinolone prescriptions in Hong Kong and 0.40 per 10,000 in the United Kingdom; notably, the incidence rate ratio was already elevated in the period before the prescription was issued, indicating substantial confounding by the underlying infection [19]. A subsequent active‐comparator new‐user cohort of 3,250,916 patients found no overall excess of seizures relative to amoxicillin (hazard ratio: 1.10, 95% CI: 0.995–1.23), with signals confined to exploratory subgroups including older adults and patients with chronic kidney disease [20]. The neuropsychiatric burden of this class is therefore better represented by delirium in hospitalized and older patients, reported in 3.7% of 631 inpatients receiving quinolone therapy for at least 48 h [21], and by peripheral neuropathy, for which a nested case–control study of 5357 cases and 17,285 controls gave an adjusted incidence rate ratio of 1.47 (95% CI: 1.13–1.92), corresponding to a small absolute excess of approximately 2.4 cases per 10,000 patients per year [22]. Pharmacovigilance data are concordant in direction, with reporting odds ratios for delirium of 4.47 for levofloxacin and 1.83 for ciprofloxacin, although such disproportionality measures have no denominator and cannot be converted into an incidence [23].

Among fluoroquinolones used in UTI management, ciprofloxacin and levofloxacin are the most commonly prescribed [24]. Ciprofloxacin, a second‐generation fluoroquinolone, has been associated with both central and peripheral nervous system toxicity. In 2013, the U.S. FDA issued a warning regarding potential neurotoxic consequences following its use [25]. CNS manifestations include seizures, encephalopathy, mania, and psychosis (Table 2).

**TABLE 2:** Case reports of levofloxacin‐ and ciprofloxacin‐induced neurotoxicity.

	Patient	Case	History	Reported adverse effect	Symptoms	Onset	Duration	Ref.
Ciprofloxacin: central nervous system side effects	78‐year‐old male	UTI	Epilepsy, seizure‐free for at least 10 years, taking only phenytoin	Seizures	Grand mal epileptic seizure	Next day following beginning of treatment	Seizures stopped once treatment was discontinued	[26]
	4‐year‐old female	UTI	Renal insufficiency, recent UTI caused by multidrug‐resistant *E. coli*	Encephalopathy	Decrease in conscious level, became comatose, hypotonic	2 h after second dose	Condition gradually improved and returned to normal 96 h after her last dose	[27]
	14‐year‐old female	Gastroenteritis	—	Antibiotic‐induced mania	Abnormal behavior (excessive laugh, irritability, elated mood)	After 2 days of treatment	Symptoms resolved 4 days after changing her antibiotics	[28]
	45‐year‐old female	Cystitis	—	Acute polymorphic psychosis with deficit in executive functions	Lack of concentration, incoherent thinking, labile and aggressive mood, nervous breakdown, concrete and incoherent thinking, auditory hallucinations and illusions	Symptoms gradually developed over 4 weeks	Patient started to improve 9 days following administration of an antipsychotic	[29]

Ciprofloxacin: peripheral nervous system side effects	42‐year‐old male	Gastroenteritis	Hypertension and diverticulitis	Axonal‐dominant polyneuropathy	Bilateral upper and lower extremity weakness	10 days after antibiotic course completion	Abnormalities persisted but significantly improved 2 months after treatment with intravenous immunoglobulin	[30]
	62‐year‐old male	UTI	—	Peripheral neuropathy	Severe numbness in the feet gradually ascending toward the calves Sensory disturbances in the hands	4 days after first intake	Patient took 6 months to recover	[31]

Levofloxacin	79‐year‐old female	Pneumonia	Hypertension, hypercholesterolemia	Delirium	Confusion, irritability, disorientation, hallucinations, impaired attention capacity	3^rd^ day of hospitalization	Condition gradually improved and delirium completely resolved 48 h after discontinuation	[32]
	68‐year‐old male	UTI	Cerebral palsy with lower limb atrophy, chronic kidney disease	Encephalopathy and myoclonus	Disturbed consciousness, difficulty with body movements, slurred speech, inability to understand complex sentences	11 days following administration	Neurotoxicity resolved following hemodialysis (through lowering blood and CSF concentrations of levofloxacin)	[33]
	58‐year‐old female	Chronic pancreatitis	No history of neurological or psychiatric illness; administered antidepressant during hospital stay	Seizures	30 s of upper‐extremity tonic–clonic contractions Loss of bowel control 2^nd^ more intense seizure after 2^nd^ dose: foaming at the mouth, rolling back of the eyes	5 h after 1^st^ dose	When antidepressant and antibiotic were discontinued, status was restored to normal	[34]

Clinical reports indicate that neurologic symptoms may develop within few hours to a few weeks after initiation of therapy, typically coinciding with active drug exposure, and often resolve rapidly following drug discontinuation.

Fluoroquinolones have additionally been linked to a spectrum of movement disorders. A recent review identified myoclonus, orofacial dyskinesia, and rapid‐onset dystonia as the most frequently reported manifestations, with ciprofloxacin implicated in the majority of cases [35]. Reports involving other agents such as pefloxacin, gatifloxacin, and ofloxacin are considerably rarer and insufficient to establish a definitive causal relationship [35].

Beyond central effects, ciprofloxacin has also been associated with peripheral nervous system toxicity, as documented in several case reports (Table 2). Notably, peripheral neuropathic symptoms tend to persist longer than central manifestations, in some cases requiring up to 6 months to resolve.

Levofloxacin, a third‐generation fluoroquinolone, has also been associated with neurotoxic effects, including acute delirium associated with psychotic features and seizures [7] (Table 2), underscoring that neurotoxicity is a class effect rather than an isolated phenomenon.

### 3.3. Carbapenems

Carbapenems are broad‐spectrum, bactericidal antibiotics belonging to the β‐lactam family and are reserved for the treatment of severe, life‐threatening infections, particularly those caused by multidrug‐resistant organisms. They are widely regarded as “last‐line” agents in critically ill patients or in settings where resistance to standard therapies is anticipated [36–39].

Like other *β*‐lactams, carbapenems inhibit bacterial cell wall synthesis by acylating penicillin‐binding proteins (PBPs), thereby preventing peptidoglycan crosslinking and leading to irreversible bacterial cell death [40]. In Gram‐negative bacteria, carbapenems enter through porin channels in the membrane before interacting with PBPs [41] (Figure 2). Due to poor oral bioavailability, carbapenems are administered intravenously and are frequently used as empiric therapy for complicated UTIs, including pyelonephritis, prior to pathogen identification [41, 42].

**FIGURE 2:**
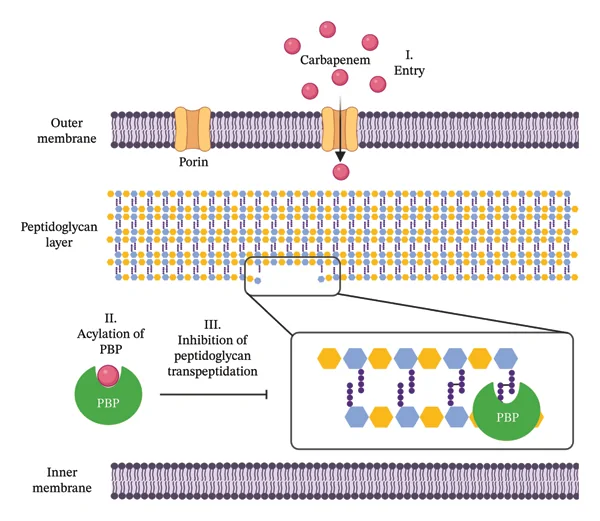
Mechanism of action of carbapenems. Carbapenems enter the bacterial cell through porins located in the bacterial membrane and acylate the penicillin‐binding protein (PBP). This enzyme is responsible for the peptidoglycan transpeptidation and formation of the peptidoglycan layer. Through its modification, peptidoglycan crosslinking is inhibited, causing bacterial cell death. This mechanism is common among all antibiotics belonging to the larger family of β‐lactams. Created with BioRender (2026).

Four carbapenems are currently approved by the U.S. FDA: meropenem, imipenem, ertapenem, and doripenem. Although all are effective against a broad range of pathogens, their neurotoxic profiles differ [43]. About 3% of patients receiving carbapenems experience seizures (mainly generalized tonic–clonic seizures), though partial seizures and encephalopathy have also been reported [7]. A meta‐analysis that included 169 studies demonstrated that the risk of carbapenem‐induced seizure was 1.87 times higher than the risk caused by other antibiotics [44].

Imipenem carries the highest relative risk of neurotoxicity among the carbapenems, although the absolute risk is low, with reported seizure rates historically quoted as ranging from 3% to 33%, particularly at higher doses. That range, however, requires careful interpretation: its upper bound derives from a single open trial of imipenem/cilastatin in 21 infants and children with bacterial meningitis, in which 7 (33.3%) developed seizure activity—a population with the highest conceivable baseline seizure risk and a disrupted BBB—and it cannot be generalized to adults receiving carbapenems for urinary or other systemic infections [45]. In contrast, doripenem and ertapenem are associated with seizure rates below 1%, while meropenem has been shown to cause continuous epileptiform discharges in approximately 0.19% of treated patients [17]. The 2004 Infectious Diseases Society of America (IDSA) bacterial meningitis guideline recommended the use of meropenem over imipenem due to the increased risk of seizures with imipenem [46]. Denominator‐based estimates are substantially lower than the historical range. In the pooled registration trials underpinning regulatory labeling, seizures were reported in 0.4% of 1723 adults receiving imipenem/cilastatin, 0.7% of 2904 patients receiving meropenem, and 0.5% of patients receiving ertapenem versus 0.3% for piperacillin/tazobactam and 0% for ceftriaxone [47]. A meta‐analysis of randomized trials found that carbapenems as a class conferred a seizure odds ratio of 1.87 (95% CI: 1.35–2.59) relative to non‐carbapenem comparators, corresponding to an absolute excess of approximately 2 seizures per 1000 patients treated; the signal was driven almost entirely by imipenem (odds ratio: 3.50, 95% CI: 2.23–5.49), whereas meropenem (1.04, 95% CI: 0.61–1.77), ertapenem (1.32, 95% CI: 0.22–7.74), and doripenem (0.44, 95% CI: 0.13–1.53) showed no significant excess [44]. It should be noted, however, that in the small number of trials comparing imipenem and meropenem directly, no difference in epileptogenicity was demonstrated, so the imipenem signal rests on indirect comparison. Observational data drawn from populations that randomized trials systematically exclude give higher and differently ordered estimates: in a retrospective series of 1345 hospitalized adults aged 60 years or older, seizures occurred in 2.4% overall, with rates of 0.7% for imipenem, 2.9% for meropenem, and 3.5% for ertapenem [48], and a separate cohort of 1706 ertapenem‐treated adults reported seizures in 1.9%, with prior stroke the strongest predictor (odds ratio: 14.36, 95% CI: 4.38–47.02) [49]. The discrepancy between trial‐derived and real‐world estimates is itself informative and reflects the exclusion of patients with epilepsy, structural CNS disease, and severe renal impairment from registration trials. A class‐by‐class comparison of penetration, mechanism, incidence, manifestations, reversibility, and risk factors is provided in Table 3.

**TABLE 3:** Comparative profile of neurotoxicity across the four broad‐spectrum antibiotic classes commonly used in the management of urinary tract infections.

	Fosfomycin	Fluoroquinolones (ciprofloxacin, levofloxacin)	Carbapenems (imipenem, meropenem, ertapenem, doripenem)	Cephalosporins (ceftriaxone, ceftazidime, cefepime)
Blood–brain barrier penetration (CSF as % of plasma, uninflamed meninges)	Paradoxically high despite marked hydrophilicity: 27 ± 8%, rising to 46% (IQR: 36–59) in ventriculitis. Small molecular size and minimal protein binding offset full ionization at physiological pH.	Highest of the four classes. Ciprofloxacin 21%–34%; levofloxacin ∼71%; ofloxacin ∼65%. Moderate lipophilicity, low protein binding, and reduced polar surface area favor passive diffusion.	Low. Meropenem ∼5%–9% (AUC ratio up to 0.39 with inflamed meninges); imipenem AUC ratio ∼0.14 (inflamed). Highly polar and ionised at physiological pH. No human CSF data exist for ertapenem (∼95% protein bound).	Low and generation‐dependent. Ceftriaxone 0.6%–1.8%; ceftazidime ∼6%; cefepime ∼8% (range 4%–34%). Third‐ and fourth‐generation agents penetrate better than earlier generations.
Principal neurotoxic mechanism	Not established. No GABA‐A antagonism has been described. A reduction in hippocampal neural stem cell proliferation has been reported in rats (preclinical only).	GABA‐A receptor antagonism (C7 piperazinyl/pyrrolidine pharmacophore, markedly potentiated by NSAIDs such as biphenylacetic acid) together with NMDA‐mediated excitotoxicity through interference with the voltage‐dependent magnesium block. Pro‐oxidant mitochondrial injury reported for ciprofloxacin.	Competitive GABA‐A receptor antagonism, determined by the basicity of the C2 side chain: imipenem (strongly basic primary amine) ≫ meropenem (weakly basic). Dominant antioxidant over pro‐oxidant effect.	Competitive GABA‐A receptor antagonism through direct receptor interaction. Potency is structure‐dependent and spans more than 160‐fold across the class by intracerebroventricular convulsant dose. Renal accumulation is the dominant clinical amplifier.
Incidence of neurotoxicity	Mild nervous‐system events only: headache 3.9%, dizziness 1.3%, asthenia 1.1% (*n* = 1233, pooled registration trials). No denominator‐based estimate of serious neurotoxicity exists; no nervous‐system signal in a combined literature and FAERS review; no neurological events in a 224‐patient real‐world intravenous cohort.	Seizures: absolute risk 0.40–0.72 per 10,000 prescriptions; no overall excess versus amoxicillin (HR: 1.10, 95% CI: 0.995–1.23) in 3.25 million patients. Delirium or psychosis in 3.7% of hospitalized patients treated for ≥ 48 h. Peripheral neuropathy adjusted IRR 1.47 (95% CI: 1.13–1.92), absolute excess ∼2.4 per 10,000 patient‐years.	Class seizure OR 1.87 (95% CI: 1.35–2.59) versus non‐carbapenem comparators; absolute excess ∼2 per 1000. Registration‐trial rates: imipenem 0.4%, meropenem 0.7%, ertapenem 0.5%. By agent: imipenem OR 3.50 (2.23–5.49); meropenem OR 1.04 (0.61–1.77); ertapenem OR 1.32 (0.22–7.74); doripenem OR 0.44 (0.13–1.53). Higher in adults ≥ 60 years (2.4% overall).	Cefepime 0.07%–0.4% by passive case‐finding (*n* = 4446); ∼1 per 480 courses; 1.0%–10% across seven cohorts; 23.2% in a cohort selected by therapeutic drug monitoring (not a population incidence). β‐Lactams in intensive care generally 2.1%–2.6%. Randomized data: delirium or coma 20.8% (cefepime) versus 17.3% (piperacillin/tazobactam).
Major neurological manifestations	Headache, dizziness, and asthenia. No established serious central nervous system syndrome.	Central: seizures, encephalopathy, confusion, delirium, psychosis, mania, and tremor. Movement disorders: myoclonus, orofacial dyskinesia, rapid‐onset dystonia. Peripheral: sensory and sensorimotor neuropathy—a class‐defining feature not shared with the β‐lactams.	Predominantly generalized tonic–clonic seizures; partial seizures; encephalopathy; myoclonus (meropenem).	Encephalopathy with altered mental status (∼93% of cefepime cases), myoclonus (∼37%), nonconvulsive status epilepticus (∼28%), seizures, aphasia, and reduced consciousness. Characteristic EEG: generalized periodic discharges with triphasic morphology.
Reversibility and time course	Events are mild and transient, resolving with completion of the (usually single‐dose) course.	Central manifestations typically develop within hours to weeks and resolve rapidly after discontinuation. Peripheral neuropathy is the exception: symptoms may persist for up to 6 months, and risk remains elevated for up to 180 days after exposure.	Generally reversible after withdrawal or dose reduction, with recovery over days; strongly associated with drug accumulation in renal impairment.	Generally reversible. Median onset 4 days; median improvement 2–3 days after dose reduction or withdrawal; approximately 90%–97% of reported patients improve. Recovery may be delayed in severe renal impairment or critical illness; dialysis is occasionally required.
Major risk factors	None established. Theoretical concern only where the blood–brain barrier is disrupted.	Older age; renal impairment (exploratory subgroup); preexisting CNS disease or epilepsy; concomitant NSAIDs (biphenylacetic acid potentiates GABA‐A blockade); concomitant antidepressants (levofloxacin); prolonged or repeated courses, with peripheral neuropathy risk rising ∼3% per additional day of exposure.	Renal impairment with failure to dose‐adjust; preexisting CNS disease or structural brain lesion; prior seizures (ertapenem OR 4.02); prior stroke (ertapenem OR 14.36, 95% CI: 4.38–47.02); older age; high dose; coadministration of cilastatin prolonging imipenem exposure.	Renal impairment is dominant, being present in 80%–90% of cefepime neurotoxicity cases, with eGFR < 30 mL/min/1.73 m^2^ the strongest independent predictor. Dosing above the renally adjusted maximum (41%–50% of cases); older age; preexisting CNS disease; critical illness; low albumin; longer duration of therapy. Trough concentration: none observed below 7.7 mg/L, ∼50% probability at ∼16 mg/L, universal at ≥ 38.1 mg/L.

*Note:* AUC, area under the concentration–time curve; GABA, γ‐aminobutyric acid. Incidence estimates are not directly comparable across classes because they are derived from different study designs, populations, and methods of case ascertainment; figures obtained from cohorts selected by therapeutic drug monitoring or by referral are identified as such in the text and should not be read as population incidences.

Abbreviations: CI, confidence interval; CNS, central nervous system; CSF, cerebrospinal fluid; EEG, electroencephalography; eGFR, estimated glomerular filtration rate; FAERS, FDA Adverse Event Reporting System; HR, hazard ratio; IQR, interquartile range; IRR, incidence rate ratio; NMDA, N‐methyl‐D‐aspartate; NSAID, nonsteroidal anti‐inflammatory drug; OR, odds ratio.

Imipenem is routinely coadministered with cilastatin, a dehydropeptidase‐1 (DHP‐1) inhibitor that prevents renal degradation of imipenem, thereby prolonging its systemic exposure. While this enhances antibacterial efficacy, it may also increase the risk of neurotoxicity through increased drug exposure [50]. Consequently, imipenem/cilastatin is contraindicated in meningitis and should be used cautiously in patients with renal impairment or preexisting CNS disease [51, 52]. Several case reports describing neurotoxic effects of imipenem and ertapenem (Table 4) have been documented.

**TABLE 4:** Case reports of imipenem‐ and ertapenem‐induced neurotoxicity.

	Patient	Case	History	Reported adverse effect	Symptoms	Onset	Duration	Ref.
Imipenem	82‐year‐old‐male	Pneumonia	Type II diabetes, angina, cerebrovascular accident (CVA)	Seizure	Tremors of the mouth and eyes, developed into generalized tonic–clonic tremors lasting 1 min	3^rd^ day of therapy	Seizures ceased after administration of phenytoin but jerking movements of the body and eyes returned 2 days later	[53]
	69‐year‐old male	Infected right knee	Head trauma and alcoholism	Seizure	Clenched fists, jerking of the head lasting 1‐2 min	7 h after dosage was increased	Another seizure occurred 4 days later but phenytoin dosage was increased, and no seizures later occurred	[53]

Ertapenem	67‐year‐old male	Spinal abscess	Chronic lumbar spine osteomyelitis, non‐Hodgkin’s lymphoma (in remission for 20 years), hypertension, atrial fibrillation, type II diabetes, ankylosing spondylitis, esophagitis	Encephalopathy	Altered mental status, hallucinations, dystonic symptoms	5 days after ertapenem was started	Mental status returned to normal 2 days after discontinuation	[54]
	84‐year‐old female	UTI	Hypertension	Encephalopathy	Confusion, irrelevant speech, inability to recognize close relatives, visual hallucinations	7^th^ day of ertapenem therapy	Patient recovered 14 days after ertapenem was stopped	[55]
	74‐year‐old male	Polymicrobial bacteremia	End‐stage liver disease, liver transplant recipient, dialysis dependent	Voice tremor and altered mental status	Tremor in his voice, weakness, confusion, forgetfulness, change in personality	1 week into therapy	Patient returned to normal rapidly after the discontinuation of ertapenem	[56]

Meropenem has been associated with rare cases of myoclonus characterized by involuntary muscle jerks [57], while reports of neurotoxicity involving doripenem remain scarce.

### 3.4. Cephalosporins

Cephalosporins are β‐lactam antibiotics that share a common mechanism of action with carbapenems, namely, inhibition of bacterial cell wall synthesis through modification of PBPs and disruption of peptidoglycan crosslinking in bacterial cells [58]. This family of antibiotics is subdivided into five generations, each with distinct antimicrobial spectra against Gram‐positive and Gram‐negative bacteria [58].

Third‐ and fourth‐generation cephalosporins exhibit the broadest activity and are widely used to treat UTIs among a wide range of infections. Analysis of the French pharmacovigilance database identified cefepime as the cephalosporin most frequently implicated in serious CNS AEs, followed by ceftriaxone and ceftazidime [59].

Third‐generation cephalosporins such as ceftriaxone and ceftazidime are commonly used in UTI management [60]; however, multiple cases of neurotoxicity have been reported, including encephalopathy, seizures, and altered mental status (Table 5). Among cephalosporins, cefepime, a fourth‐generation agent, appears to carry the highest neurotoxic risk, with numerous case reports correlating its use to severe CNS manifestations (Table 6). Quantitatively, cefepime‐induced neurotoxicity is uncommon but is consistently the most frequently reported cephalosporin‐associated CNS complication, and published estimates vary widely according to how cases are ascertained. Passive case‐finding in a retrospective review of 4446 treated patients identified suspected neurotoxicity in 0.4% and confirmed neurotoxicity in 0.07% [70], and a parallel institutional estimate gave approximately one case per 480 courses [71]. A systematic review tabulating seven cohorts reported incidences between 1.0% and 10%, the higher figures arising in intensive care and hematology populations with impaired renal function [72], whereas a cohort restricted to patients undergoing therapeutic drug monitoring (TDM) reported 23.2%, a figure that reflects preferential testing of patients already suspected of toxicity and must not be read as a population incidence [73]. In critically ill patients receiving β‐lactams generally, neurotoxicity has been documented in 2.1%–2.6% across two retrospective cohorts totaling 2090 patients [74]. Randomized evidence is limited to a single pragmatic trial in which patients allocated to cefepime had fewer days alive and free of delirium and coma than those allocated to piperacillin/tazobactam (11.9 versus 12.2 days; odds ratio: 0.79, 95% CI: 0.65–0.95), with delirium or coma occurring in 20.8% versus 17.3%; this was a secondary outcome of an open‐label single‐center trial, and the absolute difference was small [75]. Placed in context, antibiotic‐associated encephalopathy of all causes was identified in 1.0% of 97,433 hospital admissions, with β‐lactam monotherapy accounting for an incidence of 0.8% and with a clear dose–response relationship across declining categories of glomerular filtration rate [76].

**TABLE 5:** Case reports of ceftazidime‐ and ceftriaxone‐induced neurotoxicity.

	Patient	Case	History	Reported adverse effect	Symptoms	Onset	Duration	Ref.
Ceftazidime	78‐year‐old male	Pyelonephritis and right ureteral obstruction	Peripheral neuropathy, stool incontinence, benign prostatic hyperplasia, chronic urinary retention, metastatic prostate cancer to lymph nodes, lungs, bone, brain, and liver	Jerky movements of the jaw and upper extremities	Bilateral, symmetrical, myoclonic jerky movements of the jaw and arms that interfered with speaking and swallowing	On Day 3 of admission	Neurological symptoms improved drastically over the 48 h following ceftazidime discontinuation	[61]
	67‐year‐old female	Progressive respiratory failure, respiratory infection	Acute myeloid leukemia, febrile neutropenia developed during admission, acute chronic kidney injury	Encephalopathy	Hearing loss, nausea, mild confusion, progressed into hallucinations, progressive loss of consciousness, asterixis. Symptoms then evolved into myoclonic seizures and finally coma (after ceftazidime discontinuation)	6 days following the start of ceftazidime therapy	8 days following administration, ceftazidime was discontinued. Symptoms started to improve 3 days after discontinuation	[62]

Ceftriaxone	78‐year‐old female	UTI	Hypertension, ESRD, hemodialysis 3 times/week	Encephalopathy	Disturbed consciousness, drowsiness, inability to open eyes or speak	9 days following ceftriaxone administration	Patient started to improve 3 days after discontinuation, and on Day 4, her conscious level returned quickly to normal	[63]
	56‐year‐old male	Septic shock secondary to pneumonia and pleurisy	Pancreas‐kidney transplantation 5 years ago, on hemodialysis due to graft failure	Encephalopathy	Disorientation, agitation, occasional unresponsiveness to stimuli, involuntary facial movements, sporadic phonation	7 days following ceftriaxone administration	Patient completely recovered 5 days after cessation of treatment	[64]
	78‐year‐old male	Bacteremia caused by UTI	Hypertension, ischemic stroke, type II diabetes, ESKD due to diabetic nephropathy, is on maintenance hemodialysis	Encephalopathy	Deteriorating consciousness, myoclonic jerks in the legs	8 days after administration	After stopping ceftriaxone and hemoperfusion on Day 11, patient recovered	[65]
	38‐year‐old male	Enteritis	Leriche syndrome, intestinal malrotation, cholecystitis, ESKD due to chronic glomerulonephritis	Encephalopathy	Altered mental state, myoclonic leg movements	Day 4 after administration	Ceftriaxone was discontinued on Day 5 and patient underwent hemoperfusion on Day 6, after which his condition returned to normal	[65]

**TABLE 6:** Case reports of cefepime‐induced neurotoxicity.

Patient	Case	History	Reported adverse effect	Symptoms	Onset	Duration	Ref.
71‐year‐old female	Atypical pneumonia	Diabetes, liver cirrhosis, lung cancer, renal failure	Nonconvulsive status epilepticus (NCSE)	Stupor, 1 episode of brief generalized myoclonic seizure, altered mental status, minimal response to noxious pain	5th day after cefepime administration	Symptoms improved 3 days after cefepime discontinuation	[66]
64‐year‐old female	UTI	Scleroderma with pulmonary fibrosis, renal transplant recipient, pericardial effusion, hypertension, acute chronic renal transplant rejection.	Delirium	Fluctuating mental status, hysterical laughter, …	Day 6 of cefepime administration	Over the next 3 days after cefepime discontinuation, symptoms improved	[67]
88‐year‐old male	Pneumonia and sepsis	End‐stage renal disease (on dialysis), peripheral vascular disease, and gangrenous foot leading to left below the knee amputation	Encephalopathy and delirium	Myoclonic seizures in face, limbs, trunk leading to status epilepticus	Symptoms gradually developed during hospital stay	Condition improved and seizures stopped after cefepime discontinuation, antiepileptic drug administration, and increased dialysis sessions	[68]
83‐year‐old male	Malignant otitis externa	Diabetes, chronic kidney disease (CKD)	Hyperactive delirium	Not detailed	Day 5 of cefepime	Delirium resolved 3 days after switching cefepime with meropenem	[69]

Class‐specific adverse effects of antibiotics are well recognized. For example, fluoroquinolones have been associated with tendinopathy and Achilles tendon rupture [77], whereas nephrotoxicity has been reported with carbapenems, cephalosporins (particularly first‐generation agents), and fluoroquinolones [78]. Similarly, the propensity of antibiotics to induce neurotoxicity varies across classes and is influenced by differences in their chemical structure, pharmacokinetic properties, and mechanisms of action. The molecular and cellular mechanisms underlying antibiotic‐induced neurotoxicity are discussed in the following section.

## 4. Mechanisms Underlying Antibiotic‐Induced Neurotoxicity

### 4.1. Direct Neurotoxicity: CNS Penetration

To directly induce neurotoxic side effects, antibiotics need to penetrate the CNS. CNS entry is primarily regulated by two major neurovascular interfaces: the BBB and the blood–cerebrospinal fluid barrier (BCSFB). The BBB is a highly selective interface separating the systemic circulation from the CNS parenchyma. It is formed of four main cellular components: endothelial cells (ECs) connected by tight and adherens junctions, pericytes embedded within a shared basement membrane with ECs, astrocytic end‐feet, and resident microglia. Together, these cellular and molecular components maintain CNS homeostasis by restricting entry of xenobiotics and harmful pathogens and limiting inflammatory trafficking [79]. The BCSFB, while generally less restrictive than the BBB, provides complementary protection and is located at the choroid plexus within the brain ventricles. It consists of a vascular stroma containing immune cells surrounding fenestrated capillaries, overlaid by a single layer of specialized cuboidal epithelial cells; phagocytic Kolmer cells adhere to the ventricular surface. In addition to barrier function, the choroid plexus is the principal site of cerebrospinal fluid (CSF) production [80]. Under physiological conditions, BBB and BCSFB permeability is tightly regulated, substantially limiting CNS access for most compounds. CNS penetration depends on multiple physicochemical and pharmacokinetic determinants, including molecular size, lipophilicity, plasma protein binding, and the extent of transporter‐mediated influx/efflux [81]. These parameters vary across antibiotic classes and contribute to differences in neurotoxicity risk.

Fluoroquinolones, which exhibit moderate lipophilicity and relatively low plasma protein binding, can cross the BBB efficiently and have been repeatedly associated with CNS adverse effects [81]. Interestingly, CNS penetration of fluoroquinolones does not always correlate with the potential for epileptogenicity [82]. Ofloxacin, which has a 50% higher CNS permeability compared to ciprofloxacin, interestingly has had fewer cases of neurotoxicity reports than ciprofloxacin [83].

On the other hand, fosfomycin, despite its hydrophilic nature, may also reach the CNS, likely facilitated by its small molecular size and minimal protein binding, and appears to penetrate more readily than many β‐lactams [81].

As for carbapenems, imipenem demonstrates approximately 2.0–7.7‐fold higher penetration into brain tissue than meropenem and doripenem. This has been attributed to its lower molecular weight (favoring passive diffusion) and reduced affinity for organic anion transporters, resulting in slower CNS elimination and potentially prolonged exposure [81].

Moreover, cephalosporins generally exhibit low lipophilicity and limited BBB penetration, entering CSF largely via passive diffusion [84]. Notably, third‐ and fourth‐generation cephalosporins tend to show greater CNS penetration than earlier generations [85]. However, in the case of an unhealthy BBB, the penetration of antibiotics significantly increases.

Among these four antibiotic classes, fluoroquinolones demonstrate the highest intrinsic BBB permeability. This is primarily due to their relatively higher lipophilicity and lower overall polarity compared with β‐lactam antibiotics and fosfomycin, which facilitates more efficient passive diffusion across lipid membranes [86–89]. Their quinolone core contributes to a reduced effective polar surface area relative to the other classes, supporting greater CNS penetration [90]. In contrast, β‐lactam antibiotics, including cephalosporins and carbapenems, are highly polar and predominantly ionized at physiological pH due to their β‐lactam and associated functional groups. This results in poor passive membrane permeability and limited BBB penetration under normal conditions [81, 91]. Fosfomycin exhibits the lowest lipophilicity among the four classes due to its phosphonic acid moiety, which is fully ionized at physiological pH. Although its small molecular size supports some degree of diffusion, its high hydrophilicity limits lipid membrane permeability, resulting in comparatively restricted CNS penetration that is also largely dependent on BBB disruption [92–94]. Importantly, pathological disruption of the BBB can markedly increase antibiotic entry into the CNS. BBB integrity may be compromised by infection‐associated inflammation (e.g., meningitis), neurological diseases, trauma, autoimmune diseases, and aging [81, 95, 96]. Mechanistically, BBB disruption frequently involves breakdown of endothelial tight junctions, enabling increased paracellular transport of circulating substances, including antibiotics [97]. Under such pathological conditions, agents that typically exhibit limited CNS exposure, including fosfomycin, meropenem, and fluoroquinolones, may reach therapeutic concentrations in CSF [98, 99]. The magnitude of antibiotic CNS penetration is therefore determined by both barrier status and drug‐specific features such as lipophilicity and molecular size [98].

### 4.2. Direct Neurotoxicity: Neurotransmitter and Cellular Dysregulation

#### 4.2.1. GABA‐A Receptor Antagonism

Once present in the CNS, several antibiotics can directly perturb neurotransmission. A predominant mechanism described across multiple classes is antagonism of γ‐aminobutyric acid (GABA) signaling. GABA is the principal inhibitory neurotransmitter in the brain and acts via GABA‐A receptors—heteropentameric ligand‐gated chloride ion channels [100]—and GABA‐B receptors—G protein‐coupled receptors [101]. The β‐lactam ring itself has long been implicated in seizure propensity, possibly due to structural similarity to GABA [102]. In rat cortical pyramidal neurons, β‐lactams were shown to inhibit GABA‐A–induced chloride currents in a concentration‐dependent manner. Imipenem, in particular, produced competitive and dose‐dependent inhibition of these currents, reducing receptor responsiveness without fully abolishing channel activity, thereby diminishing inhibitory tone and promoting neuronal hyperexcitability and seizures [103]. Out of all carbapenems, imipenem was shown to have the highest affinity to GABA‐A receptors [104], consistent with its greater seizure liability and enhanced BBB penetration. Differences in neurotoxic potential are largely attributable to structural features, particularly the basicity of the C2 side chain and affinity for GABA‐A receptors (Figure 3). Imipenem’s higher basicity and receptor affinity confer greater epileptogenic risk, whereas meropenem’s less basic side chain is associated with lower neurotoxicity [54]. Similarly, cephalosporins such as cefepime and ceftriaxone have been shown to competitively inhibit GABA‐A receptor–mediated currents through direct interaction [105]. Fluoroquinolones may also antagonize GABAergic neurotransmission; in an experimental model using rat vagus nerve, ciprofloxacin weakly inhibited GABA receptor–mediated responses, an effect that was potentiated by the nonsteroidal anti‐inflammatory drug (NSAID) biphenylacetic acid [106]. Structural determinants further modulate fluoroquinolone neurotoxicity, with the C7 substituent—particularly pyrrolidine and piperazine moieties—implicated in adverse CNS effects [107]. Mechanistically, ciprofloxacin is believed to exert antagonistic effects on GABA‐A receptors, thereby reducing inhibitory neurotransmission and promoting CNS hyperexcitability [108]. Experimental evidence further supports these findings. In a study on female rats, the repetitive administration of high‐dose ciprofloxacin (50 mg/kg/day for 14 days) induced depression‐like and anxiety‐like behaviors, accompanied by reduced levels of serotonin and GABA, respectively [109].

**FIGURE 3:**
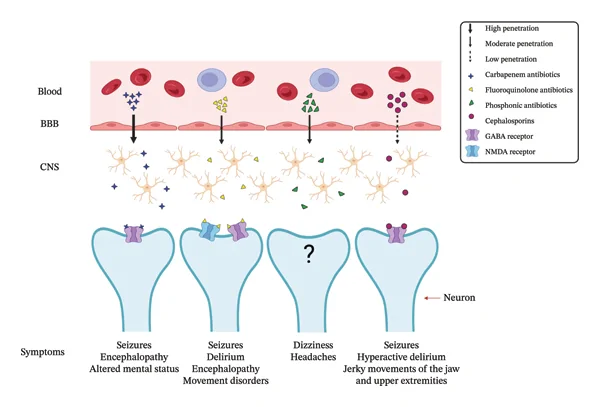
An overview of the neurotoxic potential of the most commonly prescribed broad‐spectrum antibiotic classes in the treatment of UTIs. This diagram presents a summary of the CNS penetration potential, mechanism of neurotoxicity, and the most pronounced neurotoxic side effects associated with the antibiotic classes discussed in this review. Created with BioRender (2026).

#### 4.2.2. Glutamatergic/N‐Methyl‐D‐aspartate (NMDA)–Mediated Excitotoxicity

Neurotoxicity is not solely attributable to reduced inhibitory signaling; increased excitatory neurotransmission may also contribute, often in parallel. In an in vitro rat hippocampal slice model, several fluoroquinolones increased excitatory potential, implicating modulation of glutamatergic NMDA receptors [110]. NMDA receptors are calcium‐permeable ligand‐gated channels characterized by a voltage‐dependent magnesium block that governs channel gating [111]. Fluoroquinolones are thought to interfere with this magnesium‐dependent block, thereby prolonging channel opening and increasing intracellular calcium influx, leading to heightened neuronal excitability [110]. By contrast, evidence supporting direct β‐lactam interactions with NMDA receptors is limited; their epileptogenic potential appears to be driven primarily by GABA‐A receptor antagonism as discussed above [105].

#### 4.2.3. Mitochondrial Dysfunction, Oxidative Stress, and Neuroinflammation

Accumulating evidence indicates that numerous antibiotics promote the excessive generation of reactive oxygen species (ROS) by disrupting the mitochondrial electron transport chain (ETC), resulting in oxidative damage and activation of cell death pathways [112]. Structurally and functionally impaired mitochondria exhibit increased production of ROS while generating lower levels of ATP, leading to cellular energy deficits, oxidative stress, and ultimately neuronal cell death. Evidence indicates that bactericidal antibiotics such as ciprofloxacin and kanamycin can cause mitochondrial impairment and oxidative injury in fibroblast, epithelial, and EC populations [113]. Nevertheless, the impact of these agents on human neurons is yet to be fully elucidated. Mitochondrial dysfunction is one of the leading causes of neuronal loss in several neurodegenerative disorders [114]. Neuronal survival highly depends on the integrity and full functionality of the mitochondria [115]. Therefore, mitochondrial dysfunction and neurotoxicity are highly interconnected [116].

Fluoroquinolones, in particular ciprofloxacin, are among the most frequently reported antibiotic classes associated with pro‐oxidant activity [117]. Evidently, it was shown that increased oxidative stress was implicated as a contributing factor to ciprofloxacin‐induced neurotoxicity [109]. Kalghatgi et al. measured mitochondrial superoxide production and extracellular H_2_O_2_ release in mammalian (human) MCF10A cells and found that ciprofloxacin induced a dose‐ and time‐dependent increase in both mitochondrial superoxide and H_2_O_2_. They further observed a persistent and significant increase in γ‐H2AX, a core histone protein that is phosphorylated in response to DNA damage, and 8‐hydroxy‐2′‐deoxyguanosine (8‐OHdG), an oxidized DNA byproduct, in MCF10A cells exposed to ciprofloxacin after 6 and 96 h of treatment compared with untreated cells [113]. On the other hand, cephalosporins and carbapenems have been reported to have both oxidant and antioxidant effects in different studies [117–119]. However, it has been shown that carbapenems have a dominant antioxidant over pro‐oxidant effect [120]. These differences in pro‐ and antioxidative effects among antibiotic classes directly dictate the differences in neurotoxicity elicited by them [120].

### 4.3. Indirect Neurotoxicity: Antibiotic‐Induced Gut Dysbiosis

The human gut microbiota comprises a highly diverse community of bacteria, fungi, viruses, and other microorganisms, collectively exceeding 10^14^ organisms and encoding a gene repertoire far larger than the human genome [121]. This rich ecosystem plays a crucial role in the maintenance of the body’s homeostasis; alterations in its composition and function—termed dysbiosis—have been associated not only with gastrointestinal pathology but also with systemic and neuropsychiatric consequences. A major conceptual framework linking the gut and the CNS is the gut–brain axis (GBA), a bidirectional network integrating neural (autonomic and enteric nervous systems), endocrine, immune, and humoral signaling pathways, including the hypothalamic–pituitary–adrenal (HPA) axis [122] (Figure 4).

**FIGURE 4:**
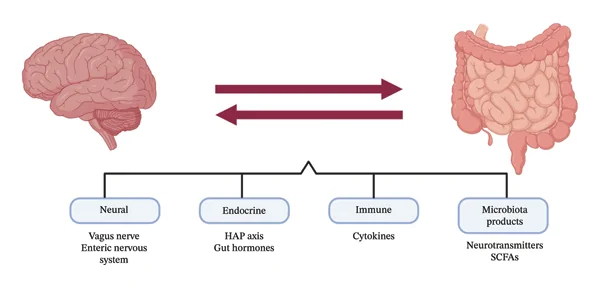
Communication pathways involved in the gut–brain axis. The gut and the brain communicate through neural pathways via the vagus nerve and enteric nervous system, endocrine pathways through the hypothalamus–pituitary–adrenal (HPA) axis and gut hormones like leptin and ghrelin, immune pathways through proinflammatory cytokines, and through products generated by the gut microbiota like neurotransmitters and metabolites including short‐chain fatty acids (SCFAs). Created with BioRender (2026).

A growing body of evidence supports microbiota‐dependent regulation of CNS function. Gut microbes play a significant role in the synthesis and modulation of neurotransmitters such as glutamate, GABA, dopamine, norepinephrine, and serotonin [123]. They also generate bioactive metabolites, most prominently short‐chain fatty acids (SCFAs) such as butyrate, acetate, and propionate [124]. SCFAs have been implicated in maintaining the integrity of the BBB and reducing its permeability, eventually exerting their neuroprotective effects. In addition, they play an anti‐inflammatory role, contribute to an increase in neurogenesis, and participate in the biosynthesis of serotonin [124]. Neurotransmitters and SCFAs are crucial in maintaining normal emotional and cognitive functions of the brain, which highlights the importance of the presence of the various bacterial species that produce them.

Microbiota‐derived signaling also interfaces with host stress and immune pathways. Intestinal bacteria can influence the HPA axis, which orchestrates the systemic stress response including cortisol release and immune system activation. Activated immune cells may release proinflammatory cytokines capable of reaching the CNS through circulation, disrupting the BBB integrity and increasing its permeability. This eventually facilitates the penetration of substances into the CNS, among which are the neuroactive or neurotoxic compounds, including certain antibiotics. In support of this, the expression of tight junction proteins at the BBB appears to be regulated by the gut microbiota [125], and reductions in tight junction components have been associated with increased BBB permeability [126], potentially altering gut–brain signaling dynamics [127].

Beyond BBB effects, the gut microbiota contributes to maintaining the integrity of the intestinal epithelial barrier. Depletion of protective, SCFA‐producing taxa may increase intestinal permeability (“leaky gut syndrome”), permitting systemic inflammation that can induce neuroinflammation and further compromise BBB function [128]. In germ‐modified mice exposed to broad‐spectrum antibiotics, downregulation of intestinal tight junction genes (*TJP1* gene encoding ZO‐1 protein, *CLDN1* gene encoding claudin‐1 protein, and *OCLN* gene encoding occludin protein) has been observed, consistent with increased intestinal barrier permeability [129].

Antibiotic exposure is a major driver of dysbiosis. Broad‐spectrum agents can markedly reduce the diversity of the gut microbiota and disrupt metabolic output [130]. The neurobiological consequences of this depletion have been characterized almost exclusively in rodents: microbiota‐depleted mice show increased motor activity with reduced vigilance and anxiety, accompanied by altered turnover of noradrenaline, dopamine, and serotonin [131]. Additional studies link antibiotic‐induced dysbiosis to impaired spatial working memory, depressive‐like behavior, and reduced exploratory activity, together with decreased levels of brain‐derived neurotrophic factor (BDNF) and altered expression of cell‐survival genes [129]. In adult C57BL/6 mice, broad‐spectrum antibiotic exposure reduced hippocampal neurogenesis, and these effects persisted beyond the treatment interval, suggesting long‐lasting effects of dysbiosis on neurogenesis [132]. Consistent with these findings, a recent study demonstrated that antibiotic‐induced dysbiosis was followed by anxiety‐like behavior, decreased BDNF mRNA expression in the ventral hippocampus, and reduced neurogenesis in the same region [133].

The strength of this evidence must, however, be stated plainly, since almost all of it is preclinical. Human data bearing on the proposed gut–brain pathway are observational, inconsistent in direction, and—critically—none of the available clinical studies measured the gut microbiota at all, so the microbiome remains an inferred rather than a demonstrated intermediary. Large database analyses have reported associations between antibiotic exposure and subsequent depression and anxiety, with a dose–response gradient across repeated courses [134], and between prolonged midlife antibiotic use and modestly lower later cognitive scores [135], an effect confirmed at meta‐analysis only for chronic and not for acute exposure and shown to be fragile on sensitivity analysis [136]. Set against these, a prospective cohort of 13,571 community‐dwelling adults aged 70 years or older—the population most closely resembling that at risk of antibiotic‐induced neurotoxicity in the context of UTI—found no association between antibiotic use and incident dementia (hazard ratio: 1.03, 95% CI: 0.84–1.25) or cognitive impairment (hazard ratio: 1.02, 95% CI: 0.94–1.11) [137], and a large matched inpatient analysis reported a reduced rather than increased risk of mood and anxiety disorders after antibiotic exposure once inflammation, comorbidity, and concomitant CNS medications were accounted for [138]. The direction of causation is itself uncertain, and at least one national claims analysis interprets the association as depression predisposing to infection rather than the converse [139].

Translation from rodent models is a further and substantial limitation. Approximately 85% of the bacterial genera found in the murine gut are absent from the human gut, and the anatomical substrate differs profoundly, with a small‐intestine‐to‐colon surface area ratio of approximately 18 in mice compared with approximately 400 in humans [140]. Germ‐free animals model the *absence* of a microbiota rather than its perturbation, and the multidrug, near‐sterilizing regimens used to deplete the rodent gut have no counterpart in a short therapeutic course for a UTI. A systematic appraisal of human microbiota–associated rodent studies found that 36 of 38 (95%) reported successful transfer of a disease phenotype from human donor to rodent recipient, a success rate the authors judged implausible and indicative of systematic overstatement of the causal role of the microbiome [141].

Most directly relevant to the present review, we are aware of no study, of any design, that has linked antibiotic‐induced dysbiosis to a neurological or neuropsychiatric complication in patients treated with antibiotics for a UTI. The two halves of this question have been examined separately but never joined. Antibiotics used for UTI demonstrably perturb the gut microbiota in exactly the population of interest: outpatients treated with ciprofloxacin showed reductions in *Bifidobacterium*, *Alistipes*, and several Firmicutes genera, whereas nitrofurantoin had minimal impact [142]. However, it should be noted that that work measured no neurological outcome. Conversely, the substantial literature on delirium in older adults with UTI has not measured the gut microbiota [143], and a recent review of the microbiota and delirium in older persons concluded that only a small number of studies have addressed the relationship at all [144]. The indirect, dysbiosis‐mediated pathway described in this section should therefore be read as a biologically plausible and mechanistically detailed hypothesis generated by preclinical work, and not as a clinically demonstrated route of antibiotic‐induced neurotoxicity. It stands in clear contrast to the direct pharmacological mechanisms described above, for which human evidence is well established—including an identified incidence of antibiotic‐associated encephalopathy of 1.0% of hospital admissions, characteristic electroencephalographic patterns, and a reproducible concentration–toxicity relationship [145].

### 4.4. Relative Contribution of Barrier Permeability, Receptor Affinity, and Systemic Exposure to Antibiotic‐Induced Neurotoxicity

Because barrier disruption increases CNS drug entry, it is tempting to treat permeability as the principal determinant of which antibiotics cause neurotoxicity. The available evidence does not support this. Permeability is best understood as a permissive condition—an agent that cannot reach the CNS cannot act there—rather than as the variable that discriminates between agents, and at least four observations argue against a permeability‐only model.

First, when the barrier is bypassed altogether, large differences in intrinsic epileptogenic potency persist. In a systematic comparison of β‐lactams administered by direct intracerebroventricular microinjection in rats, convulsant doses spanned more than a 160‐fold range within the cephalosporins alone, from cefazolin at the most epileptogenic extreme to cefoxitin at the least, and imipenem was approximately 4.4‐fold more potent than meropenem; the authors specifically noted that lipophilicity alone did not explain these differences [146]. Because these compounds were delivered directly into the ventricles, the observed hierarchy is a property of the molecules and their receptor interactions rather than of their ability to cross a barrier.

Second, penetration and neurotoxicity are dissociated, and in some pairs, they move in opposite directions. Among the fluoroquinolones, ofloxacin achieves roughly two‐ to three‐fold higher CSF penetration than ciprofloxacin (approximately 65% versus 21%–34% of plasma concentrations in patients with uninflamed meninges) [147], yet binds the GABA‐A receptor approximately 22‐fold more weakly in standardized binding assays [148]. Among the carbapenems, meropenem penetrates the inflamed BCSFB as well as or better than imipenem yet is markedly less epileptogenic both experimentally and in meta‐analyzed trial data. Fosfomycin provides the mirror image: it achieves the highest CSF penetration of the four classes considered here—27 ± 8% in critically ill patients with uninflamed meninges and 46% (interquartile range 36–59) in patients with ventriculitis [149]—while having no recognized epileptogenic signal. Cefepime completes the pattern in the opposite direction, with unremarkable penetration (approximately 8% of plasma concentrations with uninflamed meninges, lower than meropenem and far lower than any fluoroquinolone) but the highest documented burden of clinical encephalopathy among the agents reviewed here. Consistent with this, experimental work on carbapenem‐induced seizures found no correlation between CSF drug concentration and epileptogenic activity, and meningitis did not increase seizure risk [150].

Third, structural determinants of receptor interaction track neurotoxicity closely. The basicity of the carbapenem C2 side chain governs convulsant activity, with the strongly basic primary amine of imipenem conferring far greater epileptogenicity than the weakly basic side chain of meropenem [151], and the C7 piperazinyl substituent performs the analogous role in the quinolones, since its removal abolishes GABA‐A binding activity.

Fourth, and of greatest practical importance at the bedside, the variable that determines which patients develop neurotoxicity is systemic exposure rather than barrier status. Cefepime is approximately 85% eliminated unchanged in the urine, and its elimination half‐life rises from approximately 2 h with normal renal function to approximately 19 h in severe renal dysfunction [152]. Across systematic reviews, 80%–90% of patients with cefepime neurotoxicity have renal impairment and 41%–50% received doses exceeding the maximum recommended for their renal function [71, 153]. The relationship is monotonic with concentration: no neurotoxicity has been observed below a trough of 7.7 mg/L, the probability approaches 50% at approximately 16 mg/L, and symptoms were universal at concentrations of 38.1 mg/L or above, with an odds ratio of 1.06 for each 1‐mg/L increment [73, 154]. Crucially, the corresponding concentration associated with a 50% probability of neurotoxicity for meropenem is a trough above 64.2 mg/L, roughly three‐fold higher than that for cefepime, despite equal or better CSF penetration [155]. Barrier permeability cannot account for a three‐fold difference in the systemic concentration required to produce the same clinical syndrome; differences in intrinsic potency and in renal handling can.

A third axis must also be acknowledged: host CNS vulnerability. In a comparison of cefepime and meropenem in 928 patients, convulsions occurred exclusively in patients with preexisting brain disorders, and none of the affected cefepime‐treated patients had renal failure [156], and preexisting CNS disease is an independent risk factor in multivariable analyses [72].

Taken together, the evidence supports a multiplicative rather than a single‐factor model, in which the risk of neurotoxicity is the product of intrinsic pharmacodynamic potency (a structural property of the molecule), achieved systemic exposure (governed principally by renal clearance and dose appropriateness), and host CNS vulnerability, with BBB permeability acting as a permissive modulator that raises CNS exposure for a given plasma concentration without determining the rank order of risk between agents. Two caveats should be stated. Receptor affinity alone is likewise insufficient as an explanation: the reported half‐maximal inhibitory concentration of cefepime at recombinant GABA‐A receptors is in the millimolar range, far above concentrations achievable in CSF, and cefepime is paradoxically anticonvulsant in the pentylenetetrazol seizure model while being proconvulsant in others [157]. In addition, unbound rather than total drug concentrations, transporter‐mediated efflux at the choroid plexus, and the accumulation of organic acids in uremia remain inadequately characterized and may reconcile part of this discrepancy.

## 5. Clinical Risk Factors for Antibiotic‐Induced Neurotoxicity

There are several patient‐related factors that significantly influence the risk and severity of antibiotic‐induced neurotoxicity. Patient‐specific risk factors include impaired renal function, advanced age, preexisting CNS conditions, damage to the BBB, and other critical illnesses [158–160]. For example, renal dysfunction is the most prominent risk factor for cephalosporin‐induced neurotoxicity, as impaired renal clearance leads to drug accumulation and prolonged systemic exposure. This association is particularly well documented for cefepime, which is primarily eliminated via the kidneys [67]. UTIs constitute one of the top indications for cefepime (22.7%) [161]. Cefepime‐induced neurotoxicity occurs between days two and five (*n* = 29, 71%), while most resolution occurs between days one and five (*n* = 29, 85%). Like other cephalosporins, cefepime’s dosage needs to be adjusted in patients with renal impairment to avoid neurotoxicity. However, patients with renal impairments do not usually undergo changes in their drug dosage [71, 161]. Hepatic dysfunction may further exacerbate neurotoxicity by impairing metabolic clearance. Furthermore, extremes of age—especially pediatric and elderly populations—are also disproportionately affected. Additionally, a history of preexisting CNS disease, such as epilepsy or prior neurologic injury, markedly increases susceptibility to antibiotic‐induced neurotoxicity.

On the other hand, there are also medication‐related risk factors. Higher doses and prolonged use of antibiotics increase risk of neurotoxicity. Additionally, drug–drug interactions further contribute to the risk; for example, concomitant administration of ciprofloxacin with NSAIDs such as biphenylacetic acid has been shown to potentiate neurotoxic effects [106]. Similarly, coadministration of levofloxacin with antidepressants has been implicated in seizure precipitation [34].

## 6. Prognosis of Antibiotic‐Induced Neurotoxicity and Management Strategies

Antibiotic‐induced neurotoxicity may be reversible or irreversible, with prognosis depending on the antibiotic class and the risk factors described above. Available evidence suggests that antibiotic‐induced neurotoxicity is generally reversible following discontinuation or dose adjustment, with most patients demonstrating clinical improvement within a few days. Nevertheless, recovery may be delayed or incomplete in severe cases or in patients with significant risk factors such as renal impairment or critical illness. Management primarily involves early recognition of neurotoxic manifestations, prompt withdrawal or dose reduction of the offending agent, and correction of underlying precipitating factors, particularly renal dysfunction. For example, cefepime‐induced neurotoxicity is primarily managed by promptly discontinuing cefepime and switching to an alternative antimicrobial agent. Delayed recognition may result in adverse outcomes, including prolonged hospitalization, intensive care unit (ICU) admission, and increased mortality risk. In cases with impaired renal function, once it is established that neurotoxicity is caused by the antibiotic, hemodialysis or hemofiltration may be required for adequate clearance of the drug [162]. In a retrospective study by Lamoth et al., involving 30 febrile neutropenic patients, the association between plasma cefepime levels and associated neurotoxicity was analyzed. Out of the 30 cases, 6 developed neurological symptoms that improved or resolved completely with cefepime discontinuation [163, 164]. Similarly, carbapenem‐ and other β‐lactam–associated neurotoxicity is generally reversible and is strongly associated with drug accumulation in the setting of renal impairment [153]. All in all, the overall prognosis of antibiotic‐induced neurotoxicity is favorable in most cases, with recovery strongly dependent on early recognition, withdrawal of the offending agent, and correction of renal dysfunction [153].

Increased awareness of the antibiotics most frequently associated with neurotoxicity and their characteristic clinical manifestations can facilitate earlier recognition and intervention. Identifying patients at increased risk, particularly those with predisposing factors, may further support individualized preventive and management strategies. In addition, intensive hospital‐based monitoring has been shown to improve the detection of associations between antibiotic exposure and adverse neurological events. Therefore, maintaining a high level of clinical vigilance is essential for the timely identification of neurotoxic effects, enabling prompt management and improving healthcare provider awareness and education regarding these complications [7, 165].

### 6.1. TDM

Given that systemic exposure is the principal modifiable determinant of β‐lactam neurotoxicity, TDM is an attractive preventive strategy, and international consensus documents now recommend routine β‐lactam TDM in critically ill patients [166]. The rationale is strong. Concentration–toxicity relationships are reproducible across independent cohorts, with cefepime trough concentrations above approximately 20–22 mg/L conferring a substantially increased risk and concentrations of 38.1 mg/L or above associated with symptoms in essentially all patients [73, 154], and concentrations associated with a 50% probability of neurotoxicity have been derived for meropenem (trough above 64.2 mg/L) and piperacillin (above 361.4 mg/L) [155]. In a prospective cohort of critically ill septic patients, excessive β‐lactam concentrations were associated with neurological deterioration in 75.7% of patients compared with 33.3% of those without, giving an adjusted odds ratio of 10.38 (95% CI: 3.23–33.35) [167].

It must nevertheless be stated clearly that no randomized trial has demonstrated that TDM‐guided dosing reduces the incidence of neurotoxicity. The largest randomized trial of β‐lactam TDM increased pharmacokinetic target attainment approximately 2.5‐fold (37.3% versus 14.6%) yet showed no difference in delirium (odds ratio: 1.3, 95% CI: 0.4–4.8) [168]; a subsequent randomized trial of model‐informed precision dosing did not measure neurotoxicity at all, with its investigators conceding that neurotoxicity is difficult to diagnose and probably underreported in the intensive care setting [169]; and the only meta‐analytic estimate of the effect of TDM on neurological AEs, derived exclusively from observational studies, is an odds ratio of 0.86 with a confidence interval spanning 0.08 to 9.65 [170]. A separate systematic review likewise found that no study had demonstrated an association between TDM‐guided dosing and the incidence of toxicity [171]. At present, the only randomized study to list neurotoxicity prospectively as an outcome is a feasibility trial of no more than 60 patients [172].

Three practical constraints further limit what TDM can currently deliver. Proposed cefepime toxicity thresholds in the published literature span approximately 7.5–49 mg/L, so no single actionable target has been established. The best‐characterized piperacillin threshold has a specificity of 96.7% but a sensitivity of only 52.2%, so a concentration below the threshold does not exclude neurotoxicity [173]. And assay access is markedly unequal: in one national survey, β‐lactam assays were available in 41% of academic but only 6% of nonacademic hospitals [174], while in a prospective feasibility study conducted in a research‐grade setting, the median time from sampling to result was 10.9 h and results were available within two dose intervals in only 67% of cases [175].

The balanced position, therefore, is that TDM is biologically well justified and should be performed where it is available for patients at highest risk—in particular those with reduced glomerular filtration rate receiving cefepime—but that it should be regarded as a rational, mechanistically grounded practice rather than an intervention proven to reduce neurotoxicity. In settings without assay access, and as adjuncts where TDM is available, the interventions with the strongest current evidence are accurate renal function–based dose adjustment, consideration of an alternative agent in high‐risk patients, use of extended or continuous infusion regimens, and prompt electroencephalography with dose reduction or drug withdrawal when neurotoxicity is suspected. Two limits of dose adjustment alone should be recognized: approximately 26% of patients with cefepime neurotoxicity had received doses appropriate for their renal function, and approximately 10% had no renal disease at all [72, 153].

In addition to established management approaches, emerging evidence suggests that probiotics may represent a potential adjunctive strategy for reducing antibiotic‐associated adverse effects on the gut microbiome. Probiotic supplementation has been investigated as a means of preserving microbial diversity, restoring gut homeostasis, and modulating immune and neuroinflammatory pathways following antibiotic therapy. Although direct evidence supporting probiotics for the treatment of antibiotic‐induced neurotoxicity remains limited, experimental and translational studies suggest that probiotics may exert neuroprotective effects through regulation of gut barrier integrity, reduction of systemic inflammation, modulation of neurotransmitter production, and attenuation of neuroinflammatory signaling. We wish to state unambiguously, however, that this represents a hypothesis for future investigation rather than an evidence‐based clinical recommendation. Experimental and translational studies suggest that probiotics may exert neuroprotective effects through regulation of gut barrier integrity, reduction of systemic inflammation, modulation of neurotransmitter production, and attenuation of neuroinflammatory signaling, but no randomized controlled trial has evaluated a probiotic against any antibiotic‐induced neurological or neuropsychiatric endpoint—a gap noted independently in a review of the microbiota and delirium in older persons, which observed that no intervention study in older adults has yet examined delirium as an endpoint [144]. Further clinical studies are warranted to determine whether these mechanisms translate into meaningful benefits for patients with antibiotic‐induced neurological adverse effects [176]. The demonstrated clinical benefit of probiotics given alongside antibiotics is confined to gastrointestinal outcomes: a Cochrane review of 38 trials and 13,179 participants reported a substantial relative reduction in *Clostridioides difficile*–associated diarrhea, with a number needed to treat of approximately 65, but this benefit was restricted to patients at high baseline risk, with no demonstrated difference at the low baseline risk typical of patients treated for uncomplicated UTI, and the finding applies only to patients who are not immunocompromised [177]. Trials of probiotics for depression and anxiety report larger effects but derive from small studies of variable methodological quality in clinically diagnosed populations and address a different condition and causal pathway from antibiotic‐induced neurotoxicity [178]. Safety considerations are also material in the population addressed by this review: a systematic review of 93 probiotic‐associated invasive infections reported a case fatality of 19.6%, with age above 60 years, concurrent antimicrobial therapy, and *C. difficile* colitis among the factors associated with death [179], and a randomized trial of probiotic prophylaxis in predicted severe acute pancreatitis was terminated after excess mortality (16% versus 6%) and nine cases of bowel ischemia, eight of them fatal, in the probiotic arm [180]. Consistent with this, current gastroenterological guidance recommends probiotic use only within the context of clinical trials for most indications [181], and no probiotic product is approved by regulatory authorities for the prevention or treatment of any disease. Probiotic modulation of the GBA in the setting of antibiotic‐induced neurotoxicity should therefore be regarded as a direction requiring properly controlled clinical evaluation, and not as a therapy to be recommended in practice.

### 6.2. A Practical Approach to Risk Assessment, Recognition, and Management

The considerations set out above can be operationalized as a sequential clinical pathway spanning risk assessment before prescribing, recognition of neurological change during therapy, focused diagnostic workup, management, and follow‐up. This pathway is summarized in Figure 5 and set out in detail in Table 7. We emphasize that it represents a pragmatic synthesis of the evidence reviewed here together with existing expert guidance, rather than a validated decision instrument; no such algorithm has been prospectively evaluated against clinical outcomes [72, 74, 153, 182].

**FIGURE 5:**
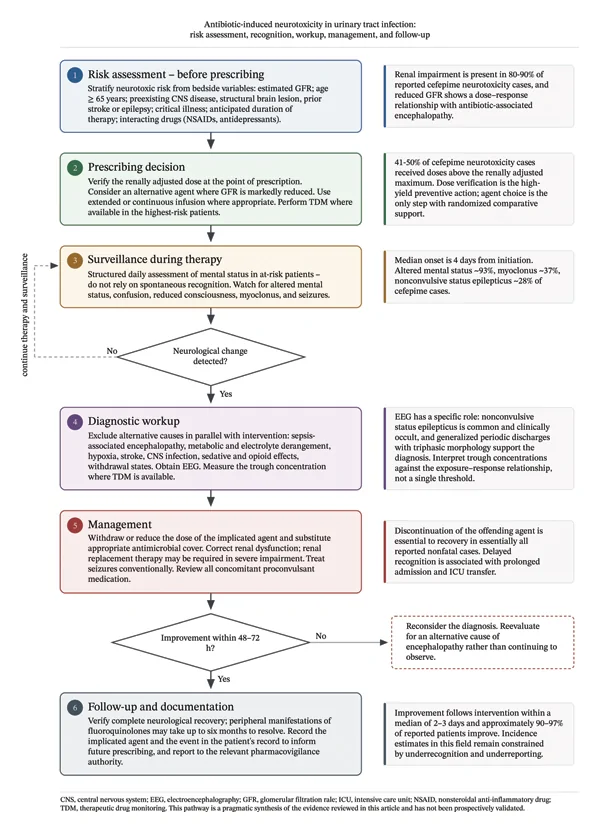
Practical algorithm for the risk assessment, recognition, investigation, and management of antibiotic‐induced neurotoxicity in patients treated for urinary tract infection. The pathway proceeds from preprescription risk stratification, through structured surveillance during therapy and focused diagnostic workup when neurological change is detected, to management and follow‐up. eGFR, estimated glomerular filtration rate; CNS, central nervous system; EEG, electroencephalography; TDM, therapeutic drug monitoring; NCSE, nonconvulsive status epilepticus. The algorithm represents a pragmatic synthesis of the evidence reviewed in this article and has not been prospectively validated.

**TABLE 7:** A practical clinical pathway for the prevention, recognition, and management of antibiotic‐induced neurotoxicity in patients treated for urinary tract infection.

Step	Action	Practical detail	Basis
1. Risk assessment (before prescribing)	Stratify neurotoxic risk using bedside variables	Estimated GFR; age ≥ 65 years; preexisting CNS disease, structural brain lesion, prior stroke or epilepsy; critical illness; anticipated duration of therapy; concomitant NSAIDs or antidepressants (fluoroquinolones)	Independent risk factors in multivariable analyses across several cohorts
2. Prescribing decision	Verify the renally adjusted dose at the point of prescription; consider an alternative agent in the high‐risk group	Renal impairment is present in 80%–90% of cefepime neurotoxicity cases, and 41%–50% received doses above the renally adjusted maximum. In patients with markedly reduced eGFR, an alternative antipseudomonal β‐lactam is a reasonable choice	Dose verification is the high‐yield preventive action; agent choice is the only intervention with randomized comparative support
3. Surveillance during therapy	Structured daily assessment of mental status in at‐risk patients; do not rely on spontaneous recognition	Median onset 4 days from initiation. Altered mental status ∼93%, myoclonus ∼37%, nonconvulsive status epilepticus ∼28% of cefepime cases. Features are indistinguishable from sepsis‐associated encephalopathy, delirium of other cause, and sedation effects	Underrecognition is the dominant limitation of the existing evidence base
4. Diagnostic workup (when neurological change is detected)	Exclude alternative causes in parallel with intervention; obtain EEG; measure the trough concentration where available	Differential diagnosis: sepsis‐associated encephalopathy, metabolic and electrolyte derangement, hypoxia, stroke, CNS infection, sedative and opioid effects, withdrawal states. EEG shows generalized periodic discharges with triphasic morphology and identifies clinically occult nonconvulsive status epilepticus. Interpret trough concentrations against the exposure–response relationship, not a single threshold	EEG has a specific role because nonconvulsive status epilepticus is common and clinically occult
5. Management	Withdraw or reduce the dose of the implicated agent; correct precipitating factors; treat seizures conventionally	Switch to an alternative agent with appropriate antimicrobial cover. Correct renal dysfunction; renal replacement therapy may be required for clearance in severe impairment. Review all concomitant proconvulsant medication	Discontinuation is essential to recovery in essentially all reported nonfatal cases
6. Confirm the diagnosis by response	Reassess at 48–72 h	Improvement follows intervention within a median of 2–3 days, and approximately 90%–97% of reported patients improve. Failure to improve within this window should prompt reconsideration of the diagnosis rather than continued observation	The time course is well characterized and is diagnostically useful
7. Follow‐up and documentation	Verify complete neurological recovery; document and report	Peripheral manifestations of fluoroquinolones may take up to 6 months to resolve. Record the implicated agent and the event in the patient’s record to inform future prescribing. Report to the relevant pharmacovigilance authority	Incidence estimates in this field are constrained by underrecognition and underreporting

*Note:* eGFR/GFR, (estimated) glomerular filtration rate. This pathway is a pragmatic synthesis of the evidence reviewed in this article together with existing expert guidance. It has not been prospectively validated against clinical outcomes and is not intended to substitute for institutional protocols or specialist advice.

Abbreviations: CNS, central nervous system; EEG, electroencephalography; NSAID, nonsteroidal anti‐inflammatory drug.

Before prescribing cefepime, risk assessment should prioritize renal function, older age, critical illness or ICU admission, and preexisting CNS disease [3–6]. However, the evidence supporting individual predictors remains predominantly observational and is not entirely consistent. Prior seizure history alone was not associated with an increased risk of cefepime neurotoxicity in a matched cohort study, while treatment duration was not significantly associated with neurotoxicity in a recent meta‐analysis [72, 182, 183]. Renal dysfunction was present in 80% to nearly 90% of patients in published systematic reviews, and dosing above renal‐adjusted recommendations occurred in approximately 41%–48% [72, 153]. Verification of renal function and the corresponding dose at the time of prescription is therefore a key preventive measure, although neurotoxicity can occur despite appropriate renal adjustment [72, 153]. When an effective alternative is available, avoiding cefepime may be reasonable in patients at particularly high risk. In the ACORN randomized clinical trial, cefepime was associated with more neurological dysfunction than piperacillin–tazobactam; however, the trial was not restricted to patients with substantially reduced glomerular filtration rates and did not specifically adjudicate cefepime‐induced neurotoxicity [75].

During therapy, the principal clinical manifestation requiring recognition is a change in mental status. In a systematic review, altered mental status occurred in approximately 93% of reported cases, myoclonus in 37%, and nonconvulsive status epilepticus in 28%, with a median onset of 4 days after cefepime initiation [72]. These manifestations may be difficult to distinguish clinically from sepsis‐associated encephalopathy, delirium due to other causes, or the effects of sedative medications [72, 153]. Regular assessment using a validated mental‐status or delirium‐screening instrument should therefore be considered in at‐risk and critically ill patients rather than relying solely on spontaneous recognition of neurological deterioration [184].

When cefepime neurotoxicity is suspected, metabolic, infectious, structural, and pharmacological causes of encephalopathy should be investigated while intervention proceeds in parallel. Electroencephalography has an important role because nonconvulsive seizures may be clinically occult [72, 153, 185]. Generalized periodic discharges with triphasic morphology are frequently reported in cefepime neurotoxicity but are nonspecific and should be interpreted in conjunction with renal function, drug exposure, clinical timing, and exclusion of competing diagnoses [186]. Where available, measurement of the cefepime trough concentration can provide supportive evidence of excessive exposure. Nevertheless, published concentration thresholds vary, and results should be interpreted within the overall exposure–response relationship rather than against a single universal toxic threshold [72, 73].

Management centers on prompt discontinuation of cefepime, or dose reduction when continued treatment is considered essential, together with correction of precipitating factors, particularly impaired renal clearance [187]. Seizures should be treated according to established clinical protocols, while hemodialysis may be considered in severe or prolonged cases, particularly in patients with advanced renal dysfunction [187]. Approximately 89%–98% of reported patients improve following intervention, generally within a median of two to three days [72, 153]. Clinical improvement after cefepime withdrawal supports causal attribution but does not by itself confirm the diagnosis. Failure to improve within the expected interval should prompt reassessment for competing or additional causes, while recognizing that delayed recovery has been documented, particularly in patients with severe renal impairment [188].

Follow‐up should confirm complete neurological recovery because resolution may be delayed or incomplete in some patients [188, 189]. In the broader context of antibiotic‐associated neurotoxicity, peripheral neurological manifestations—particularly fluoroquinolone‐associated peripheral neuropathy—may persist considerably longer than acute central manifestations and can occasionally be permanent [190]. The implicated antibiotic and adverse neurological event should be clearly documented in the patient’s medical record to inform future prescribing. Suspected cases should also be reported to the relevant pharmacovigilance authority because estimates of the incidence and burden of antibiotic neurotoxicity remain constrained by underrecognition and substantial underreporting of ADRs [191, 192].

## 7. Conclusion: Limitations and Future Perspectives

### 7.1. Limitations

Despite growing evidence linking broad‐spectrum antibiotics to neurotoxicity, the overall quality of available data remains variable. Clinical evidence is derived predominantly from case reports, case series, and retrospective observational studies, which are susceptible to selection bias, confounding, and underreporting [153]. The diagnosis of antibiotic‐induced neurotoxicity is further complicated by the presence of comorbid conditions, renal dysfunction, and concomitant medications that may independently contribute to neurological manifestations. While some studies adjusted for relevant comorbidities and patient characteristics, their findings should be interpreted cautiously, as residual confounding may persist. Factors such as varying degrees of renal dysfunction, underlying cognitive impairment, sepsis‐associated encephalopathy, and polypharmacy are often difficult to fully control for and may independently influence the development and severity of neurological symptoms. Consequently, prospective clinical studies employing standardized diagnostic criteria and long‐term follow‐up are needed to better define the incidence, risk factors, and prognosis of antibiotic‐induced neurotoxicity. The evidence assembled in this review is also heterogeneous in kind, and we have therefore indicated throughout which statements rest on clinical data, which on case reports and case series that carry no denominator, and which on in vitro or animal work alone (Table 1). This distinction matters particularly for the indirect, microbiota‐mediated pathway, which is supported almost entirely by preclinical evidence, and for several incidence figures in common circulation whose original denominators are either very small or absent.

While animal and in vitro studies have provided valuable mechanistic insights, their translational relevance is limited by interspecies differences, the use of supratherapeutic antibiotic concentrations, and simplified experimental conditions [193]. For example, the current evidence suggesting a potential effect of fosfomycin on hippocampal neurogenesis is based predominantly on experimental animal studies. Given the lack of clinical confirmation in humans, the neurotoxic significance of these findings remains uncertain and should be interpreted cautiously until validated by well‐designed clinical studies.

### 7.2. Future Perspectives

Broad‐spectrum antibiotics commonly used in the treatment of UTIs—including fosfomycin, fluoroquinolones, and β‐lactams such as carbapenems and cephalosporins—have been associated with a spectrum of neurological adverse effects, ranging from seizures and psychosis to peripheral neuropathy, movement disorders, and encephalopathy. Mechanistically, these manifestations may arise through (i) direct CNS exposure with disruption of inhibitory and excitatory neurotransmission (notably via GABA‐A antagonism and, for fluoroquinolones, modulation of NMDA receptor activity) and (ii) indirect pathways mediated by antibiotic‐induced gut dysbiosis, which can alter neuroimmune signaling, compromise intestinal and BBB integrity, and alter the neurotrophic regulation of cognition and affect.

Although antibiotic‐associated neurotoxicity remains relatively uncommon and causality can be difficult to establish in individual cases, the identification of consistent risk factors emphasizes the need for proactive mitigation strategies. Host factors—including renal dysfunction, advanced age and extremes of age, preexisting neurological disease, and relevant drug–drug interactions—should direct antibiotic selection, dosing, and monitoring. Personalized approaches have been proposed, including clinical algorithms that incorporate patient comorbidities and pharmacokinetic risk to guide safer prescribing [7]. Close clinical surveillance for early neurological symptoms during treatment is essential to enable prompt recognition, discontinuation or de‐escalation of the offending agent when appropriate, and timely supportive management.

Future work should aim to decipher specific mechanisms, define incidence across contemporary prescribing patterns, and establish evidence‐based clinical guidance. Key priorities include the development of standardized screening and monitoring protocols for high‐risk groups, improved pharmacovigilance strategies, and consensus‐based management pathways. From a translational perspective, rational modification of antibiotic structures to reduce CNS receptor interactions and neurotoxic liability—without compromising the antimicrobial activity—may represent a promising research direction. In parallel, interventions targeting microbiome resilience may prove valuable as well. Experimental studies suggest that probiotic administration following antibiotics can partially reverse dysbiosis‐associated neurobehavioral changes [129], and emerging approaches such as fecal microbiota transplantation have shown promise for restoring microbial ecology in select contexts [194]. Ultimately, judicious antibiotic use, coupled with patient and public education, remains central to minimizing both neurotoxicity and the broader public health threat of antimicrobial resistance.

## Author Contributions

Conceptualization: Lama Hachem and Wassim Abou‐Kheir; data curation: Lama Hachem, Maram Chaaban, Rawan Khadra, Hashem Shehade, Batoul Darwish, Antoine Abou Fayyad, Hisham F. Bahmad, Georges Daoud, and Wassim Abou‐Kheir; formal analysis: Lama Hachem, Maram Chaaban, Rawan Khadra, Hashem Shehade, and Batoul Darwish; investigation: Lama Hachem, Maram Chaaban, Rawan Khadra, Hashem Shehade, and Batoul Darwish; methodology: Lama Hachem, Maram Chaaban, Rawan Khadra, Hashem Shehade, and Batoul Darwish; project administration: Antoine Abou Fayyad, Hisham F. Bahmad, Georges Daoud, and Wassim Abou‐Kheir; resources: Antoine Abou Fayyad, Hisham F. Bahmad, Georges Daoud, and Wassim Abou‐Kheir; software: Antoine Abou Fayyad, Hisham F. Bahmad, Georges Daoud, and Wassim Abou‐Kheir; supervision: Hisham F. Bahmad, Georges Daoud, and Wassim Abou‐Kheir; validation: Hisham F. Bahmad, Georges Daoud, and Wassim Abou‐Kheir; visualization: Hisham F. Bahmad, Georges Daoud, and Wassim Abou‐Kheir; writing–original draft: Lama Hachem, Maram Chaaban, Rawan Khadra, Hashem Shehade, Batoul Darwish, and Hisham F. Bahmad; and writing–review and editing: Antoine Abou Fayyad, Hisham F. Bahmad, Georges Daoud, and Wassim Abou‐Kheir. All authors critically revised and edited the manuscript.

## Funding

This research received no external funding.

## Disclosure

All authors have read and approved the final draft.

## Conflicts of Interest

The authors declare no conflicts of interest.

## Data Availability

The original contributions presented in the study are included in the article; further inquiries can be directed to the corresponding authors.
